# Health Tract, No. 225

**Published:** 1864-10

**Authors:** 


### HEALTH TRACT, No. 225.

### "DIRTY CHILDREN."

There is an undefined impression left on the minds of many in passing a group of chubby looking children playing in the street or by the roadside, bare footed, bare headed and ragged, begrimed with dust or mud, that "dirt must be healthy." And when there is noticed around the cabins of the country poor or the shanties in the city outskirts a crowd of ragamuffin urchins of all sizes, like the regular gradations of a ladder, another notion is almost formed, in distinct words, that "poverty is healthy" as well as dirt, as the having a house full of children is taken as proof of vigorous constitutions on the part of the toiling parents. Taking New York City as a guide, the official reports for 1863 show that of every ten deaths, seven are foreign, although just half the population is foreign born ; and as a class, foreigners are the poorest and the filthiest of all American large seaboard cities ; of course there are notable exceptions. It is known that those who live on their daily wages average eleven years less of life than those who are well to do. So that poverty is as far from being healthful, as it is from being agreeable. Of 1000 children dying under one year old, nearly three-forths were born of foreign parents ; two-thirds of all the children dying on the day of their birth were of foreign parentage. Of those dying from one to five years old, three-forths were born of poor people. Of nine children, Queen Victoria lost none. The constitutions of Royal pairs may not be as vigorous as those of two young laborers, but exemption from exhausting toil, and their ability to command roomy residences, well ventilated chambers, and the strictest personal cleanliness from earliest infancy, more than counterbalances other unfavoring circumstances. So far then from poverty and filth being elements of health and long life, they are the very reverse ; they directly induce premature death as to grown up persons, and sow the seeds of fatal diseases in innocent childhood. During the first week of August, 1864, in New York City 444 children died ; of which, 404 were of foreign parentage, and only forty were born of native parents ; that is, ninety per cent of the children dying in New York, nine out of ten are from the abodes of poverty and untidiness.

